# Immunosenescence and Novel Vaccination Strategies for the Elderly

**DOI:** 10.3389/fimmu.2013.00171

**Published:** 2013-06-28

**Authors:** Michael G. Dorrington, Dawn M. E. Bowdish

**Affiliations:** ^1^Department of Pathology and Molecular Medicine, McMaster University, Hamilton, ON L8S 4L8, Canada

**Keywords:** immunosenescence, elderly, pneumonia, influenza, human, vaccination, vaccination strategies, adjuvants

## Abstract

Vaccination remains the most effective prophylactic intervention for infectious disease in the healthcare professional’s toolkit. However, the efficacy and effectiveness of vaccines decrease with age. This becomes most apparent after an individual reaches 65–70 years old, and results from complex changes in the immune system that occur during aging. As such, new vaccine formulations and strategies that can accommodate age-related changes in immunity are required to protect this expanding population. Here, we summarize the consequences of immunosenescence on vaccination and how novel vaccination strategies can be designed to accommodate the aging immune system. We conclude that current vaccination protocols are not sufficient to protect our aging population and, in some cases, are an inefficient use of healthcare resources. However, researchers and clinicians are developing novel vaccination strategies that include modifying who and when we vaccinate and capitalize on existing vaccines, in addition to formulating new vaccines specifically tailored to the elderly in order to remedy this deficiency.

## Introduction

Vaccination represents a cost-effective and efficient way to protect people from morbidity and mortality due to infections and, in addition, reduces hospitalizations, and the economic cost of lost productivity (Whitney et al., 2003; Jefferson et al., 2005). Since infections often accelerate or exacerbate pre-existing health concerns in the elderly, prevention of infection by vaccination facilitates healthy, independent living. Current vaccines and vaccination strategies are effective at protecting healthy adults and the vulnerable young; however, they are less effective or even completely ineffective in the elderly. This has become a growing concern for public health, especially in developed nations where high birthrates in the mid-twentieth century, medical advancement, and increasing standards of care have contributed to a ballooning elderly population (Chen et al., 2009).

As we age, changes occur in both the innate and adaptive immune compartments. This phenomenon, termed immunosenescence, increases our susceptibility to some, but not all, infectious diseases. While there have been numerous advances in our understanding of immunosenescence over the past 5 years, there has yet to be a corresponding increase in availability of novel therapies and vaccines tailored for our aging population. In this review, we will discuss the growing literature on the aging immune system, and how this information is being utilized in the design of new vaccines and vaccination strategies to protect the elderly against a variety of pathogens.

Please note that this review does not focus entirely on the mechanisms of immunosenescence. Those who wish to view a more in-depth review of these mechanisms are encouraged to read the following: Montecino-Rodriguez et al. (2013) (for overview), Jenny (2012) (inflammation), Frasca and Blomberg (2011) (B cells), and Arnold et al. (2011) (T cells).

## Recent Findings in Immunosenescence

Immunosenescence is defined as age-associated changes in the immune response. There is a general misconception that the immune system of the elderly becomes hypo-responsive or broadly non-functional; however, although many aspects of immunity decline, this does not occur uniformly and some elements of the aging immune response are preserved (e.g., CD8+ T cell polyfunctionality) (Lelic et al., 2012) while others are enhanced (e.g., pro-inflammatory cytokine production by macrophages) (Olivieri et al., 2013). Consequently, some researchers suggest that the term immunosenescence should be replaced with the phrase “senescent immune remodeling” to better describe the plasticity of the aging immune system (Dewan et al., 2012). Whatever terminology used, it is clear that aging of the immune system begins at the level of the hematopoietic stem cell (HSC), resulting in the increased susceptibility to infectious disease and decreased efficacy of vaccination in the elderly.

**Figure 1:**
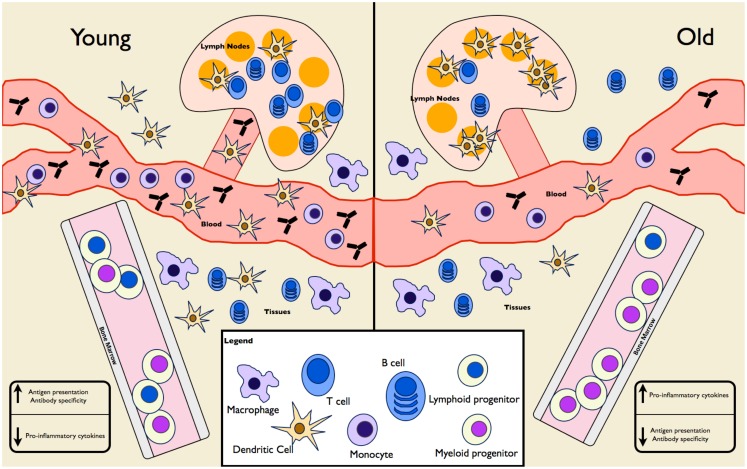
**Age-associated changes in immune cell frequency and function**. A mixture of mouse and human experiments show differences in both the frequency and function of cells in the innate and adaptive immune compartments. HSCs in the elderly are skewed toward differentiation into myeloid progenitors at the expense of lymphoid progenitors. Elderly individuals have fewer circulating monocytes and DCs, fewer tissue-associated DCs, normal tissue macrophages, and increased splenic DCs. Naïve T and B cells populations are diminished, though mature-like T and B cells are expanded. As a result, overall antibody production and specificity is diminished in the elderly. Despite increased numbers of splenic DCs, antigen presentation is lacking at these sites in the elderly. Pro-inflammatory cytokine levels are increased in the elderly, as seen in IL-6 and TNF levels in serum samples.

Hematopoiesis is the mechanism of creating blood cells by the differentiation of HSCs. With age, the proliferative capacity of these cells, as well as their ability to engraft, decreases and there is a decrease in lymphoid precursors, which contributes to skewing toward myeloid precursors (reviewed in Van Zant and Liang, 2012). The reasons for this are not clear but it appears to be a combination of epigenetic changes, DNA damage, telomere shortening, and exposure to chronic age-associated inflammation (Tollervey and Lunyak, 2012; Tumpel and Rudolph, 2012). The end result is fewer circulating lymphocytes (of particular importance is the decrease in naïve T cells) (Sun et al., 2012), increased susceptibility to anemia (due to decreased output of erythrocyte precursors) (Van Zant and Liang, 2012), and increased pyogenic bacterial infections (due to malfunctioning leukocytes) (Dewan et al., 2012). An overview of age-related changes in leukocytes is presented in Figure 1.

### Age-associated changes in innate immunity/antigen presentation

Recent evidence demonstrates that changes in the myeloid progenitors result in fewer circulating monocytes (Verschoor et al., 2013), as well as changes in the number of circulating dendritic cells (DCs) (Verschoor et al., 2013). Age-related changes in tissue resident myeloid cells are not as clear-cut. The numbers of tissue-associated macrophages are not believed to be diminished and in some studies were increased, as were splenic CD8^−^ DCs (Wong et al., 2010), whereas Langerhan’s cells, and plasmacytoid DCs are decreased (Xu et al., 2012). In addition to numerical changes in antigen presenting cells (APCs), there are significant functional changes, which include changes in surface receptor expression (Pereira et al., 2011), the ability to migrate to local lymph nodes (Grolleau-Julius et al., 2008; Zhao et al., 2011), hyper- or hypo-inflammatory responses depending on the stimuli, and a reduced ability to become fully activated (Paula et al., 2009; Mahbub et al., 2012). There is conflicting evidence as to whether antigen presentation and T cell proliferation are altered with age *in vitro* (Pereira et al., 2011; Tan et al., 2012); however, *in vivo* models demonstrate that antigen presentation and T cell proliferation are reduced (Pereira et al., 2011; Zhao et al., 2011), likely due to the reduced migration of DCs to the lymph nodes and changes in co-stimulatory receptor expression (Li et al., 2012). Some of the changes in APC function appear to be due to changes in the aging microenvironment (e.g., the increased levels of prostaglandin D2 in the aging lung reduce DC activation and expression of chemokine receptors required for them to migrate to the lymph nodes; Zhao et al., 2011), whereas other defects appear to be independent of the microenvironment (Paula et al., 2009).

Recent studies have shown that the inflammatory functions of various myeloid cell populations are increased with age, causing chronic inflammation throughout the body (Dewan et al., 2012). While the reasons for this are up for debate, researchers have consistently found higher levels of tumor necrosis factor (TNF) and various pro-inflammatory interleukins (ILs) in the serum of elderly individuals when compared to young controls (Franceschi et al., 2007). There are numerous theories as to why this occurs, including increased leakiness in the gut leading to high levels of endotoxin reaching the blood (Meier and Sturm, 2009), chronic viral infections (such as *Cytomegalovirus*) (McElhaney et al., 2012), and increased lifespan of tissue cells such as macrophages (Harper et al., 2010). This chronic inflammation, termed “inflamm-aging” has negative consequences on innate immune responses including reduced cytokine production by myeloid cells upon stimulation with toll-like receptor (TLR) ligands and reduced antigen presentation (Olivieri et al., 2013). Overall, innate immunity is abnormal in the elderly, leading to muted immune responses to pathogens and less efficient transition to adaptive responses.

### Age-associated changes in adaptive immunity

Vaccine efficacy is most frequently assessed by quantitating the levels of circulating antibodies, or by assessing the opsonophagocytic capacity of induced IgG. There is some debate about whether the quality or quantity of antibodies produced with age are decreased (Blomberg and Frasca, 2011; Sasaki et al., 2011); however, most studies consistently demonstrate that the elderly produce fewer antibodies in response to influenza and *Streptococcus pneumoniae* vaccination and this reduction correlates with decreased vaccine efficacy (Cadeddu et al., 2012). In addition, elderly individuals vaccinated with a “booster” vaccine against influenza produced less IgG than young controls, thus showing a reduction in secondary immune responses to the pathogen (Matsushita et al., 2012). These phenomena may be due in part to defects in APCs or T cell help, but changes in the distribution and numbers of B cell subsets also contribute. It is unclear whether total B cell numbers change with age; however, there is a clear-cut decrease in the numbers of naïve B cell and their precursor populations and a corresponding increase in memory-like B cells (Ongradi and Kovesdi, 2011). This is due in part to output by HSCs and myeloid skewing and possibly defects in emigration from the bone marrow. To counterbalance the effects of fewer new B cells being formed, mature B cells in the periphery tend to have increased lifespans as well as a greater proclivity to homeostatic expansion. As a result, the B cell population becomes more homogenous and less antigen-specific over time. Studies in humans have shown a relative decrease in memory B cell populations in elderly patients, possibly accounting for decreased antibody production upon secondary vaccination with influenza virus. However, some memory responses appear to remain intact, such as those stimulated by the tetanus vaccine, although these responses are dependent on pre-booster antibody levels (Hainz et al., 2005).

Similar to B cells, there are fewer naïve T cells and numbers of memory T cells increase with age and this is at least partially due to reduced output of lymphoid precursors from the bone marrow. Education in the thymus is also impaired since thymic involution, which begins at birth, is complete by age 50. As a result, the output of naïve T cells is greatly reduced. Homeostatic proliferation in the periphery might maintain the numbers of T cells; however, these cells have a limited T cell receptor (TCR) repertoire, thus reducing their ability to mount responses to novel antigens.

The end result of thymic involution and longer-lasting peripheral T cells is a population shift from predominantly naïve T cells to T cells with a memory phenotype, especially in the CD8+ T cell population, which are less capable of clonal expansion upon TCR stimulation. Recent work has shown that despite a lack of naïve CD8+ T cell numbers in the elderly, the functionality of CD8+ T cells during acute and chronic viral infections does not correlate with age. As such, CD8+ T cell-mediated responses appear to remain intact even in individuals as old as 85 (Lelic et al., 2012). While more studies need to be performed on specific T cell subsets and their abilities to mount appropriate memory responses to a wide range of pathogens needs to be performed, it appears that vaccines targeting cell-mediated adaptive immune responses might be more efficacious in the elderly than those eliciting antibody responses. It will be interesting to determine whether CD8+ T cells in the elderly are able to mount appropriate responses to intracellular bacteria, as chronic viral infection often leads to an exaggerated population of virus-specific memory T cells in older individuals.

The antigenic diversity of the CD4+ T cell population in the elderly decreases suddenly and at a greater rate than that of CD8+ T cells, although these cells appear to be more functional than their CD8+ counterparts. The numbers of CD4+ cells in the periphery is relatively stable; however, much like CD8+ T cells, there is a population shift to a memory phenotype. Little is known about how this shift affects responses to specific vaccine contents. It is postulated that due to the widespread reduction in the antigen-reactive T cell repertoire, novel antigens will have less opportunity to induce a protective memory response. As such, work has begun on “piggy-backing” vaccines in order to establish immunity to novel antigens while boosting previous vaccinations.

## Current Vaccination Programs

Due to the high social and economic costs of infectious disease in the elderly, most developed countries provide a comprehensive vaccine program to individuals over the age of 65. These programs involve both one-time booster and annual vaccinations against common, vaccine-preventable pathogens such as influenza virus and *S. pneumoniae*. Recently, the value of some of these vaccines has been called into question since, in some cases, they may not prevent disease in this population (Cadeddu et al., 2012). Consequently, it has been proposed that the best strategy for protecting the elderly might require developing new vaccines, modifying existing vaccines, or changing current vaccination practices to more effectively evoke herd immunity.

### Influenza

Severe influenza virus infections are very common in the elderly and are often accompanied by fatal secondary bacterial infections. As such, it is generally recommended that everyone over the age of 65 receive the seasonal trivalent inactivated influenza vaccine (TIV) as well as pandemic influenza vaccines, if applicable, in order to maintain serum antibody levels. The effectiveness of this strategy is debatable, with multiple studies of the efficacy of the vaccine in the elderly being performed with little consensus between studies (Prelog, 2012). This lack of consensus is due to a lack of study protocol standardization and outcome parameters, as well as insufficient consideration of patient frailty and study bias. A number of meta-analyses have shown a reduction in influenza-specific hospitalizations by 27–45% and death by up to 50%, compared to 70–90% efficacy in healthy, young adults (Gross et al., 1995; Jefferson et al., 2005; Manzoli et al., 2012). Thus, based on the data that has been collected, it can be concluded that the efficacy of the influenza vaccine is reduced in individuals over the age of 65 and is negatively correlated with age within this cohort (Chen et al., 2009).

It is generally believed that the reduction in influenza vaccine efficacy in the elderly is linked to multiple defects in the immune response to the vaccine including reduced anti-hemagglutinin (HA) antibody titers as well as reduced specificity of these antibodies (Sasaki et al., 2011). The reasons for these diminished responses are due to a reduced heterogeneity in the B cell pool brought about by the long-term maintenance of memory B cells and reduced production of B cell precursors in the bone marrow, as noted earlier. Interestingly, the initial inflammatory response to the vaccine also differs depending on age. For example, elderly individuals produce elevated serum IL-6 levels, a hallmark of the innate immune response (Trzonkowski et al., 2009). As such, it appears that both the innate and adaptive immune responses to the influenza vaccine are affected by age, though the specific changes require further research.

### Streptococcus pneumoniae

There are currently two forms of pneumococcal vaccine available. The conjugate pneumococcal vaccines (e.g., Prevnar^®^ 13 and SynFlorix) consist of tetanus, diphtheria, and non-typeable *Haemophilus influenzae* carrier proteins conjugated to capsular polysaccharides of the 13 (Prevnar^®^) or 10 (SynFlorix) most common strains found in children (Canada, 2012). Immunization with these strains induces antibodies that clear nasopharyngeal carriage and protect from invasive pneumococcal infection and has dramatically reduced pneumococcal infections in children and indirectly in the elderly due to a reduction of circulating strains (see Section Herd Immunity). Since adults, and especially the elderly, become infected with a broader range of serotypes and are able to mount anti-polysaccharide antibody responses, the 23-valent polysaccharide vaccine (Pneumovax^®^ 23 or Pneumo 23^®^) are currently recommended (Canada, 2012). Unlike the conjugate vaccine, immunization with the polysaccharide vaccine does not induce robust mucosal immunity and, thus, does not result in reduced colonization or provide herd immunity; however, it has been shown to reduce mortality due to invasive pneumococcal disease in healthy young adults (Butler et al., 1993). In the elderly, however, pneumococcal pneumonia is a far more common manifestation of disease and mortality than is invasive pneumococcal disease (Jokinen et al., 1993). Although immunization of the healthy elderly might reduce invasive pneumococcal disease, there is little or no protection from mortality due to pneumonia in either the healthy or frail elderly (Dear et al., 2003; Johnstone et al., 2010; Cadeddu et al., 2012). Better immunization strategies are required to protect the elderly from pneumonia, which is a leading cause of mortality and a contributor to decreased quality of life in this population.

### Diphtheria, tetanus, and pertussis

Despite waning levels of diphtheria, tetanus, and pertussis infection worldwide, these diseases have not been eradicated, with the elderly having the highest incidence of infection (Chen et al., 2009). Pertussis, in particular, remains at consistent levels in this population, though cases are severely underreported (Tan et al., 2005). The tetanus, diphtheria, and acellular pertussis (Tdap) vaccine is recommended for all individuals over the age of 65. A small number of studies have been performed in order to track what proportion of elderly individuals have protective antibody titers, with the results being disconcerting. According to Alagappan et al. (1997), 33% of elderly study participants had protective levels of anti-pertussis antibodies and that 68% of these patients were not protected against tetanus. The efficacy of Tdap boosters in elderly patients relies heavily on pre-vaccination antibody titers, with higher antibody titers before boosting correlating with positive vaccine responses. Currently, most vaccines that are suggested for the elderly are not being given at any point between childhood and the age of 65. It might be that in order to increase the boosting ability of vaccines later in life, people should receive boosters intermittently throughout their lifetimes, before immunosenescence detrimentally affects vaccine responses. This will ensure that antibody titers (and other measures of vaccine efficacy) are at optimal levels and could improve boosting immunizations later in life. If antibody levels are too low, elderly individuals are unable to mount appropriate responses and previous immunizations will not have lasted. This should be taken into account when public health officials are recommending vaccine schedules.

## Novel Approaches to Vaccination in the Elderly

**Figure 2:**
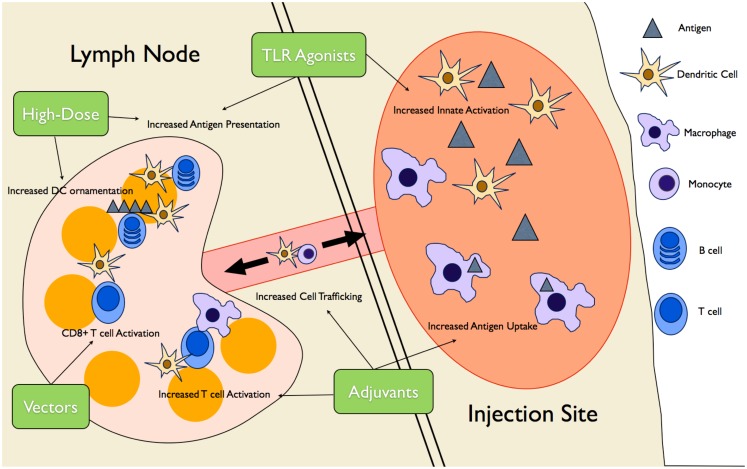
**Novel vaccine interventions in the elderly**. Multiple novel vaccination strategies are being tested to boost the vaccine response in the elderly. High-dose vaccines increase antigen presentation as well ornamentation of follicular DCs with antigen complexes, increasing B cell activation over time. Various adjuvants (MF59, AS03, and Matrix-M™) create an “immunocompetent” environment at the injection site, increasing trafficking of DCs and monocytes to and from the site, increasing antigen uptake and presentation to T cells. TLR agonists are used to increase innate immune cell activation and, therefore, antigen presentation. Finally, viral vector vaccines increase CD8+ T cell responses by promoting antigen presentation on MHCI.

The current lack of vaccines approved for use in the elderly, combined with the limited efficacy of current vaccines in this population, has led researchers to attempt to generate vaccines aimed at individuals over the age of 65. This is being done by either utilizing available vaccines that are not currently approved for use in the elderly, or else generating novel vaccine strategies targeting the aberrant immune responses described earlier in this review. These strategies can be as simple as increasing vaccine dosage or as complex as introducing viral vector-based vaccines to combat disease. As mentioned above, the vast majority of this research is being conducted in order to combat influenza, a fact that is reflected in the remainder of this review. Figure 2 provides an pictorial overview of the intervention strategies currently being researched in the elderly as well as the aspects of the immune response they are meant to target.

It is important to note that the studies outlined below suffer from the same lack of standardization as the efficacy studies explained in Section “Recent Findings in Immunosenescence.” Of particular interest is how researchers define immunogenicity when testing novel vaccination methods. In the case of influenza, most studies focus on the generation of systemic antigen-specific antibodies or opsonophagocytic ability of these antibodies. However, it has been shown that the activity of T cells (and especially cytotoxic T cells) might be of equal importance for protection (McElhaney et al., 2006; Wilkinson et al., 2012). As such, measures of immunogenicity should include measures of T cell activation.

### High-dose vaccines

The best studied, and simplest, approach to increasing vaccine efficacy in the elderly is increasing the dose given. Interestingly, modification of antigenic dose seems to require a tailored approach to each age group as in infants low-dose vaccines are more effective at generating CD8+ T cell responses (Sarzotti et al., 1996). In the elderly, high-dose vaccines are used to increase antigen delivery from APCs, such as DCs, to B cells, eliciting a greater antigen-specific antibody response. Immunization with high-dose vaccines is believed to increase the numbers of follicular DCs ornamented with antigen–antibody immune complexes. These cells are capable of stimulating B cell responses in the absence of new antigen for long periods of time (El Shikh et al., 2010). A high-dose TIV (60 μg of HA per strain vs. 15 μg given normally) has reached phase IIIb clinical trials with mixed results. One double-blinded, randomized, multi-center trial in adults over 65 years of age showed a slight decrease in influenza incidence in those immunized with the high-dose vaccine when compared to low-dose controls (DiazGranados et al., 2013). The increase in protection of 12.6% is misleading, however, as the 95% confidence interval ranged from −140.5 to 65.8%, showing high variability in the results. A similar phase III immunogenicity trial showed that protective antibody titers were significantly higher in individuals given high-dose vaccine and that these individuals were also more likely to seroconvert 28 days after vaccination when compared to those receiving a standard-dose TIV (Falsey et al., 2009). These effects were even seen in individuals over the age of 75. There were, however, no measurements of vaccine efficacy in regard to influenza incidence or morbidity, thus limiting the scope of the trial. While increasing the dose of TIV might increase the immunogenicity of the vaccine, it remains to be seen whether these increases warrant a change in vaccine policy for elderly individuals.

While tests have been performed on the efficacy of the high-dose influenza vaccine, few other vaccines have been tested in this manner. The conjugate pneumococcal vaccine has recently been approved for use in individuals over the age of 65. A study in 2011 showed that increasing the dosage of the vaccine would increase efficacy without increasing the rate of adverse events in subjects who were 70 years old or older. In this study, when patients were given a double dose of PCV7 and PCV9, there was a dose-dependent increase in local cytokine production and opsonophagocytic activity of serum antibodies, as well as systemic antibody titers when compared to single doses of the two vaccines (Lode et al., 2011). The study reported that negative reactions to the two vaccine formulations were comparable. Perhaps more importantly, the effects of the double dose conjugate vaccine were significantly greater (for the serotypes used) than the 23-valent polysaccharide vaccine that is given most regularly to this age group. While more studies need to be performed to assess the long-term benefits of the high-dose vs. low-dose conjugate vaccine as well as the benefits of these vaccines compared to the polysaccharide vaccine (and whether these benefits outweigh the high cost of manufacture for the conjugate vaccines), the work that has been done shows that increasing the dose of the current vaccines on the market might make them better suited to the elderly population.

### Adjuvants

With dose increases of vaccines currently in use leading to mixed results, many researchers have turned to the alteration of vaccine contents, including the addition of novel adjuvants. A number of potential TIV adjuvants are being tested in mice, with a few additional candidates in clinical trial or already in use. The method of action of these adjuvants is most commonly based on stimulating enhanced innate immune responses, and especially the influx and activation of APCs at the site of vaccination (Tetsutani and Ishii, 2012). This increase in APC activation leads to more robust adaptive responses and greater immunological memory. In the elderly, innate immune responses are often enhanced compared to adaptive responses, making this arm of immunity promising for exploitation by vaccine adjuvants.

The first adjuvant in use in TIV, MF59, has been included in influenza vaccines for the elderly in parts of Europe since 1997 and was recently licensed in Canada for individuals over the age of 65. MF59 is an oil-in-water emulsion that creates an “immunocompetent environment” at the injection site, leading to increased cellular infiltration, especially by APCs, in a CCL2/CCR2-dependent manner. This, in turn, amplifies the amount of antigen being presented to T and B cells (O’Hagan et al., 2012). Due to its widespread use in elderly populations in Europe, there is considerable data on the safety and immunogenicity of the MF59-adjuvanted vaccine. Elderly individuals vaccinated with this vaccine consistently respond with high antibody titers and increased rates of seroconversion, which result in higher protection. Post-immunization reactions are more frequent in those receiving the adjuvanted vaccine, but are tolerable. Due to variability in the design of efficacy studies, there is some controversy as to whether the adjuvanted vaccine provides better protection than regular TIV, although multiple controlled studies show efficacy greater than 50% (Czajka et al., 2012; Della Cioppa et al., 2012; Fukase et al., 2012; Mannino et al., 2012).

A second adjuvant, AS03, is currently in phase IIb clinical trials. AS03 is very similar to MF59 in that both are predominantly made up of squalene in an oil-water emulsion. As such, it is believed to function in a similar manner to that of MF59, although the mechanism of action has not been as rigorously determined. A recent trial in elderly individuals showed a somewhat increased relative efficacy when compared to non-adjuvanted TIV (McElhaney et al., 2013). However, this study also suffered from wide variability as seen in the 95% confidence intervals (12.11%, −3.40 to 25.29), despite being a very large study (43,802 participants). AS03 also suffers from allegations of causing narcolepsy in young children who have received pandemic influenza vaccines containing the adjuvant (Miller et al., 2013). While these effects have not been seen in the elderly in safety trials, the negative effects seen in children could make it difficult to license any vaccine using AS03 as an adjuvant, especially when it is so closely related to MF59, which has shown to be safe in numerous trials.

Matrix-M™ (AbISCO^®^ -100, Isconova AB, Sweden) is a saponin-based vaccine adjuvant that has been tested (in the context of TIV) in mice and has reached Phase I trials in elderly humans in the past year. Matrix-M™ functions similarly to AS03 and MF59, increasing cellular trafficking to the site of injection and stimulating T cell immunity (Reimer et al., 2012). Matrix-M™ has been shown to induce both Th1 and Th2 responses in mice, unlike the common adjuvant alum, which induces only Th2 responses. A recent study performed in mice showed Matrix-M™ to be a superior adjuvant to AS03, alum, and Freund’s Complete Adjuvant (FCA) (Magnusson et al., 2013). Mice who received Matrix-M™ had more cells traffic to draining lymph nodes and the spleen, as well as a higher proportion of fully mature macrophages, DCs, and NK cells at the site of injection. Perhaps most importantly, there was a significant increase of activated CD4+ and CD8+ T cells in the draining lymph nodes. The researchers then went on to use Matrix-M™, alum, and AS03 in a vaccine with inactivated influenza virus antigens. Mice received one of unadjuvanted vaccine or vaccine together with Matrix-M™, AS03, or alum. After 45 days, spleens were collected and splenocytes were stimulated with the relevant antigens. Splenocytes from mice receiving Matrix-M™ along with vaccine produced significantly higher pro-inflammatory cytokine levels, including IL-2 and interferon (IFN)-γ. IgG responses in these mice were similar to mice receiving AS03 as an adjuvant. This study shows that saponin-based adjuvants such as Matrix-M™ might provide increased protection over other adjuvants that are already in use, or are undergoing clinical trials now.

Numerous animal studies have been performed regarding TLR agonists as adjuvants (reviewed in Duthie et al., 2011). It is believed that it might be possible to increase the activation of the adaptive immune response by taking advantage of the overactive innate immune response in elderly individuals. However, it has been found that the adjuvant activity of TLR agonists can be improved upon using fusion proteins (Fujita and Taguchi, 2012). One of the most promising candidates in this regard is the TLR5 agonist flagellin. When flagellin is administered intratracheally to mice, aged mice respond better than young controls as measured by innate immune cell trafficking to draining lymph nodes and T and B cell activation (Bates et al., 2008). However, IgG and IgA production in the aged mice did not reach the same levels as young controls. Despite this, the aged mice given flagellin did produce significant amounts of these antibodies, possibly highlighting a way to increase vaccine immunogenicity. Animal studies have also been performed using flagellin-based fusion proteins with pneumococcal surface proteins and conserved influenza virus proteins, with excellent results, though these studies were conducted in young adult mice, not aged (Skountzou et al., 2010; Nguyen et al., 2011).

Recently, a recombinant HA-flagellin fusion protein influenza virus vaccine (called VAX128) has been tested in healthy adults as well as individuals over the age of 65. VAX128 is designed to increase immunogenicity to pandemic strains of influenza virus while limiting the adverse effects that are often associated with these potent vaccines. Safety tests in humans have shown VAX128 to be well tolerated by the elderly, even at relatively high dosages (Taylor et al., 2012). More exciting are the immunogenicity data from these trials. Anti-HA antibody titers, as well as seroconversion and seroprotection in the elderly participants reached levels comparable to young controls and were greater than those found in individuals vaccinated with TIV. T cell-specific responses were not measured, however. While much more work will be required in order to show the efficacy of vaccines based on TLR agonists, initial results are very encouraging.

### Vector-based vaccines

Recent work has shown that CD8+ killer T cell responses against various viruses remain intact in the elderly (Lelic et al., 2012). However, the bulk of our current vaccines target the humoral response mediated by CD4+ helper T cells and plasma cells. In order to exploit aspects of adaptive immunity that remain intact throughout the aging process, researchers are turning to vector-based vaccines. Recently, studies have been performed using Modified Vaccinia virus Ankara (MVA) as a vector for highly conserved influenza proteins nucleoprotein (NP) and matrix protein 1 (M1). This vaccine, MVA-NP+M1, improves upon TIV in two ways: it targets CD8+ T cell responses and does so using antigens that are common to all strains of influenza, thus eliminating the need for the serotype matching needed to generate effective TIV vaccines each year. Antrobus et al. (2012) performed safety and immunogenicity studies in volunteers over the age of 50 using MVA-NP+M1 with mixed results. The vaccine was tolerated well by all of the participants, with very few adverse events recorded. Vaccine immunogenicity was measured by measuring the number of NP- or M1-specific CD8+ T cells producing IFN-γ in the blood. While individuals between the ages of 50 and 69 responded very well to the vaccine, those over the age of 70 were unable to sustain T cell numbers at a protective levels 3 weeks after vaccination. Interestingly, the researchers detected NP- or M1-specific CD8+ T cells that also produced TNF-α and IL-2 in all of the age groups, showing that the cells produced were polyfunctional, an important characteristic of robust immune responses to T cell antigens (Antrobus et al., 2012). Much like the flagellin recombinant vaccine, studies on MVA-NP+M1 are in their very early stages. However, there is enough evidence to show that these vaccines might be effective in the elderly population and so they will likely enter later-stage clinical trials in the near future.

### Herd immunity

Until the aforementioned challenges in vaccinating the elderly are overcome, the best protection might be in relying on the herd effect to protect them indirectly. Herd immunity occurs when the percentage of individuals vaccinated is high enough that the rate of disease decreases to the point where the probability of unvaccinated individuals contracting disease is minimal (Kim et al., 2011). In essence, the pathogen is kept at a subclinical level in the population as a whole protecting those who are at risk of infection. If this approach is to be taken to protect the elderly from infections in which vaccination is only efficacious in the young, current vaccination strategies must be altered significantly. For example, during seasonal influenza infection, most countries target vulnerable populations in whom the death rates are highest (i.e., the elderly, pregnant women, individuals with chronic diseases). Some also target health care workers who come into contact with these individuals although vaccination rates among these individuals often remain too low to be protective (Thomas et al., 2010). Combined these vaccinations strategies may prevent some deaths in these vulnerable groups, but they are sub-optimal for preventing infections in the elderly for two reasons. The first is that vaccine efficacy is lower in the elderly. Even in years in which the influenza vaccine is well matched and efficacious in young people, efficacy in the elderly can be<20% (Prevention, 2013). Until these efficacy rates are improved by novel vaccination strategies, prevention of infection, rather than vaccination is required. The second reason is that the attack rate (i.e., the cumulative incidence of infection during an epidemic) in the elderly is considerably lower in the elderly than adults or children (9.3–13.5 per 100 persons ≥65 years, compared to 20–40 per 100 persons in children ≤9 years) (Glezen, 1996). It is estimated that as many as 50% of the cases of influenza in the elderly are caused by exposure to grandchildren (Towers and Feng, 2012) and that grandparents who are caregivers for young children have extremely high rates of influenza and pneumonia (Cohen et al., 2011a). Consistent with this observation, both influenza and pneumococcal vaccination of children have been demonstrated to reduce the incidence of infection, hospitalization, and deaths in the elderly (Monto et al., 1969; Whitney et al., 2003; Hsu et al., 2009; Loeb et al., 2010; Cohen et al., 2011b). In addition, indirect measures of controlling infections in children (e.g., school closures) have a pronounced effect of decreasing disease rates in the general population, including the elderly (Earn et al., 2012). Although vaccinating children for influenza or pneumonia is predicted to be a cost-effective measure (Salo et al., 2006), when the indirect effects of vaccination are taken into account the cost-effectiveness of vaccination (as measured by the cost per life-year saved) increases as much as 15-fold (Ray et al., 2006; Pitman et al., 2013).

## Conclusion

Improving the quality of current vaccination programs for the elderly will require advances in our understanding of how the immune system ages as well as how we can exploit those changes to induce more robust, long-lasting responses to vaccine contents. While these advances will take some time to come to fruition, there are many policy decisions that need to be made in the meantime in order to better protect our aging population. Altering vaccine schedules and targeting at-risk populations are two ways in which we can improve vaccine efficacy without the need for novel vaccines to come to market. The vaccination of the elderly is a perfect example of where researchers (developing new vaccines), clinicians (testing new and current vaccines), and other healthcare professionals (prescribing and administering vaccines) need to work together in order to ensure complete vaccine coverage in this population as well as data collection and analyses in order to pinpoint areas of need. Vaccine development would benefit from more standardization of efficacy trials in regards to both current and novel vaccine approaches.

## Conflict of Interest Statement

The authors declare that the research was conducted in the absence of any commercial or financial relationships that could be construed as a potential conflict of interest.
